# The economic and health burden of stroke among younger adults in Australia from a societal perspective

**DOI:** 10.1186/s12889-021-12400-5

**Published:** 2022-02-03

**Authors:** Elise Tan, Lan Gao, Janice M. Collier, Fiona Ellery, Helen M. Dewey, Julie Bernhardt, The AVERT Trial Collaboration Group, Marj Moodie

**Affiliations:** 1 Deakin Health Economics, Institute for Health Transformation, Deakin University, Geelong, Australia; 2 Florey Institute of Neuroscience and Mental Health, University of Melbourne, Heidelberg, Australia; 3 Eastern Health Clinical School, Monash University, Box Hill, Australia

**Keywords:** Young, Stroke, Economic, Societal, Burden

## Abstract

**Background:**

To estimate the short term (5 years) and long term (30 years) economic burden of stroke among younger adults (18–64 years), and to calculate the loss of health-related quality of life in these individuals, in Australia.

**Methods:**

A Markov microsimulation model was built to simulate incidence of stroke among younger adults in Australia. Younger adults with stroke commenced in the model via health states defined by the modified Rankin Scale at 12 months from the AVERT study (A Very Early Rehabilitation Trial), and transitioned through these health states. Costs in Australian dollars (AUD) were measured from a societal perspective for a 2018 reference year and categorised into medical, non-medical and indirect costs. Probabilistic sensitivity analyses were performed to test the robustness around the cost of illness estimates. The loss of health-related quality of life due to stroke among younger adults was calculated by determining the difference in estimated quality-adjusted life years (QALYs) between the stroke population and the general population. This was determined by multiplying the predicted remaining life years for the modelled stroke cohort and the age-matched general population, by their corresponding age-dependent utilities.

**Results:**

The economic burden of stroke among younger adults was estimated to be AUD2.0 billion over 5 years, corresponding to a mean of $149,180 per stroke patient. Over 30 years, the economic impact was AUD3.4 billion, equating to a mean of $249,780 per case. Probabilistic sensitivity analyses revealed a mean cost per patient of $153,410 in the short term, and a mean cost per patient of $273,496 in the long term. Compared to the age-matched general population, younger adults with stroke experienced a loss of 4.58 life years and 9.21 QALYs.

**Conclusions:**

The results of our study suggests high economic and health burden of stroke among younger adults and highlights the need for preventive interventions targeting this age group.

**Trial registration:**

ACTRN12606000185561, retrospectively registered.

**Supplementary Information:**

The online version contains supplementary material available at 10.1186/s12889-021-12400-5.

## Background

Stroke is a leading cause of death and disability in Australia and worldwide [1]. It is often thought of as an older person’s disease – but people of any age can suffer a stroke. Approximately 25% of first-ever strokes in adults occur in those under the age of 65 [2]. Though people of working age represent a small proportion of total stroke patients, they carry a disproportionately larger share of the resultant economic burden [3].

Some 65% of stroke survivors live with debilitating effects and experience reduced quality of life, resulting in significant costs and productivity losses [4]. In Australia, approximately 30% of stroke survivors are aged under 65 years, equating to an estimated 149,000 people in 2018 [4, 5]. As younger survivors live with the health consequences of stroke for longer, and stroke related costs are sustained over an extended period, the associated economic and health burden is distinct from that of stroke in the elderly.

Several Australian studies have investigated the economic impact of stroke, however, none were specific to persons of working age. The total lifetime cost of stroke in Australia was estimated as Australian dollars (AUD) 3.1 billion in 2010 (US dollars (USD) 2.1 billion), corresponding to a total cost per case of AUD99,938 [6]. A Very Early Rehabilitation Trial (AVERT) provides recent comprehensive resource use information relating to the 12 months following a stroke, and an opportunity to estimate the impact of stroke among younger adults (18–64 years) in Australia. Participants were included in this trial as being disabled post stroke and being able to benefit from rehabilitation. This examination is of interest to policy makers and consumer groups alike, by informing future policy decisions, resource allocation and priority setting for stroke. Using modelling of data, the aim of this paper is to determine 1) the economic (medical, non-medical and indirect) costs of stroke among younger adults (18–64 years); and 2) the associated loss of health-related quality of life.

## Methods

### Cost of illness

A Markov microsimulation model [7] was constructed to estimate the economic burden of stroke among younger adults in Australia. The model evaluated the cost of illness over the short term (5 years) and the long term (30 years) time horizons. The quality of health economic studies (QHES) checklist was used to guide the methodological quality of our study [8].

#### Model structure

**Fig. 1:**
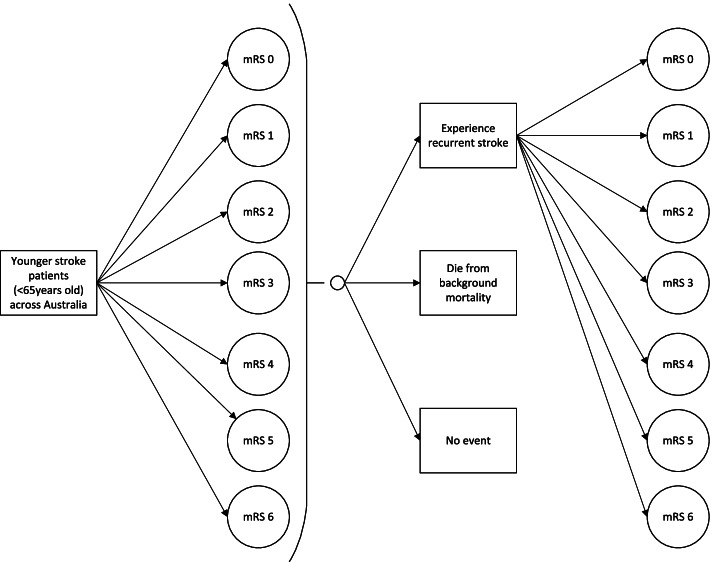
Structure of the cost of illness microsimulation model. Notes. The assumption is that a patient’s health state cannot improve. *mRS* modified Rankin Scale

The structure of the model is depicted in Fig. 1. Microsimulation was used to simulate the passage of each hypothetical patient through the model following time-dependent state transition probabilities. The model captured all seven health states associated with functionality post stroke, defined by the modified Rankin Scale (mRS) ranging from a mRS score of 0 (no symptoms) to 6 (dead). Following on from their initial health state (12 months post stroke), survivors may experience recurrent stroke events where they could either die or transition to the same or a worse health state, die from background mortality, or have no event (i.e. remain in the same health state). The model was developed in TreeAge Pro 2019, R2 (Treeage Software Inc., Williamston, Massachusetts, USA).

#### Population

Stroke cases among younger adults in Australia in 2018 were simulated. Patient characteristics were based on data from the Australian Institute of Health and Welfare (AIHW) and were assumed to be representative of the Australian younger adult stroke population – the median age fell between the 45–54 age group, and 60.07% were male [9]. Consistent with the national stroke audit, it was assumed that 82% of strokes were ischaemic and 12% were intracerebral haemorrhage (remaining 6% were undetermined) [10].

The modelled population was calculated based on the proportion of younger patients with stroke (23.58%) out of the incidence of all stroke events (first-ever and recurrent stroke across all ages) [9]; this equates to 13,555 cases of the estimated 57,487 cases of stroke that occurred in Australia in 2018 [5].

#### Model inputs

Where appropriate, model inputs were drawn from AVERT, described elsewhere [11]. In brief, this was a multinational study examining outcomes for patients who received very early mobilisation in addition to usual care compared to usual care alone. Resource use (medical and nonmedical such as hospitalisations and community services), short-term transition probabilities, and health-related quality of life outcomes for the first 12-months were sourced from patient-level data from the Australian arm of the AVERT study. In AVERT, resource use questionnaires were administered in person and collected by trained staff at 3 and 12 months post stroke. Model parameters outside this timeframe were obtained from published literature. Model inputs are described below and primary inputs are presented in Table 1.

**Table 1:** Model inputs, varied by mRS score

**Probability and utility inputs**			
**State**	**mRS probability (range**^**a**^**)**	**Short term utility**^**b**^ **(range**^**a**^**)**	**Long term utility** [12–14] **(range**^**a**^**)**
mRS 0	0.1654 (0.1489–0.1820)^b^	0.85 (0.76–1.00)	0.85 (0.80–1.00)
mRS 1	0.2964 (0.2668–0.3261)^b^	0.78 (0.67–0.94)	0.80 (0.75–0.90)
mRS 2	0.2551 (0.2296–0.2806)^b^	0.67 (0.53–0.89)	0.70 (0.53–0.75)
mRS 3	0.1379 (0.1241–0.1516)^b^	0.30 (0.12–0.42)	0.51 (0.45–0.65)
mRS 4	0.0276 (0.0248–0.0303)^b^	0.11 (0.02–0.20)	0.30 (0.25–0.55)
mRS 5	0.0310 (0.0279–0.0341)^b^	0.03 (0.00–0.07)	0.15 (0.00–0.32)
mRS 6	0.0866 (0.0779–0.0953) [9]	–	–
**Short term annual cost**^**b**^**, AUD**			
**State**	**Medical (range**^**a**^**)**	**Nonmedical (range**^**a**^**)**	**Indirect (range**^**a**^**)**
mRS 0	15,464 (5685-26,522)	570 (0–1140)	14,733 (0–32,196)
mRS 1	21,901 (6106-39,353)	2112 (0–4224)	27,400 (0–50,593)
mRS 2	40,408 (14,837-51,458)	5236 (140–6460)	47,534 (0–87,389)
mRS 3	68,199 (44,273-68,953)	30,821 (13,133-40,867)	36,357 (0–87,389)
mRS 4	85,578 (71,406-103,792)	58,569 (29,341-74,610)	43,301 (0–91,988)
mRS 5	140,089 (71,464-161,818)	82,999 (46,339-70,034)	40,781 (0–87,389)
mRS 6	43,644 (3918-42,359)	19,738 (0–2627)	39,612 (0–82,789)
**Long term annual cost, AUD**			
**State**	**Medical** [6, 15] **(range**^**a**^**)**	**Nonmedical** [6, 15] **(range**^**a**^**)**	**Indirect** [16] **(range**^**a**^**)**
mRS 0	1400 (0–2800)	576 (0–1152)	–
mRS 1	1400 (0–2800)	576 (0–1152)	2998 (0–5996)
mRS 2	1774 (0–3548)	730 (0–1460)	19,855 (0–39,710)
mRS 3	1774 (0–3548)	730 (0–1460)	27,875 (0–55,750)
mRS 4	13,715 (0–27,430)	5648 (0–11,296)	43,301 (0–86,602)
mRS 5	17,545 (0–35,090)	7225 (0–14,450)	40,781 (0–81,562)
mRS 6	–	–	–

*mRS* modified Rankin Scale, *AUD* Australian dollar

^a^The range used for sensitivity analyses

^b^Derived from AVERT trial patient level data

##### Transition probabilities

All simulated patients started in the model in one of the health states defined by the mRS outcome at 12 months, as observed in AVERT (missing data: 3.50%). Stroke population one-year stroke recurrence rate obtained from a meta-analysis of studies worldwide (including Australia), was 2.01% per year [17]. Though this included all age groups, this was comparable to rates observed in individuals under 50 years [18]. The model assumed no recurrence during the first year as the costs and change in outcome due to recurrent stroke were already captured from the 12-month follow up. Age dependent mortality for recurrent stroke was used as the risk of death varies with age [19] – the AIHW reported mortality of 26.83% for patients 65 years and over, and 8.66% for those under 65 was used [9]. After accounting for death, the model assumed the distribution of possible mRS health states following recurrent stroke was equal. The model was adjusted for the greater risk of recurrent strokes amongst persons with a previous history of stroke. A risk ratio of 1.39 was applied, along with a multiplicative nature with each previous event [20]. The general population mortality rate was calculated using the age and gender-dependent death rates in Australia from 2016 to 2018 [21].

##### Costs

A societal perspective was adopted to measure all the costs related to stroke. All costs are reported in 2018 AUD. USD equivalents are provided based on 2018 exchange rates (1 AUD = 0.784USD) [22]. Any dated prices were inflated using the health price index specified by the Australian Bureau of Statistics [23]. Future costs were discounted at a 5% rate [24].

A range of costs were considered, grouped via the following categories: medical, nonmedical and indirect costs. Twelve-month resource use data were drawn from AVERT. Relevant unit costs were then applied to determine the mean costs associated with each health state [25].

Medical costs included costs of acute hospitalisation and stroke-related re-hospitalisations; ambulance transport; rehabilitation; and respite care. All hospitalisation costs were derived from the Independent Hospital Pricing Authority (IHPA) [26–28]. Ambulance transport was costed using a median cost based on all Australian states [29–31]. Outpatient rehabilitation costs were sourced from Comcare compensation data [32], whilst overnight respite costs were sourced from the Aged Care Financing Authority [33]. The costs of medication or general practitioner (GP) visits were not collected as part of AVERT.

Nonmedical costs included costs of changes in accommodation; community services; home modifications; equipment and aids; and informal care (care provided by family or friends). Changes in accommodation were costed only where the patient transitioned from home to a nursing home or hostel following stroke [33]. Costs of home modifications and equipment/aids were obtained from a range of product catalogues, and informal care was valued in accordance with the minimum wage for paid carers [34].

Indirect costs from lost productivity were calculated using a human capital approach, where any hour of potential working life not worked due to illness was costed. This was based on the change in work hours from pre stroke to 1 year post stroke (based on change from baseline to 3 months and then 3 months to 12 months), valued using average age-based earnings in Australia [35].

Long term management costs (i.e. 12 months post stroke) were obtained from published literature. Direct medical (aged care facilities, medication, community services, inpatient rehabilitation, GP care, hospitalisations and other direct medical costs) and nonmedical (caregiver out of pocket costs and informal care) costs were based on a 10-year Australian longitudinal study [6], stratified by mRS score weights derived from an average cost analysis [15]. The model adjusted for the return to work rate following the index stroke for each health state (i.e. mRS score) to estimate the productivity loss accumulated by patients of working age who did not return to the workforce [16].

##### Utility

Effectiveness was measured by quality-adjusted life years (QALYs), determined by the number of years lived multiplied by the utility score for being in particular health state(s). Utility scores, derived by measuring preferences for particular health states, can be viewed as preference weights. Both total QALYs across Australia and mean QALYs per case are reported.

Utility scores, valued between 0 (dead) and 1 (perfect health), assigned to each health state (mRS score) enabled the calculation of QALYs. Short term utility scores were based on the mean utility for each health state in AVERT which were measured based on the Assessment of Quality of Life (AQoL)-4 dimension (4D; collectively AQoL-4D) collected at 12 months using Australian population preferences. Long term utility scores were sourced from published literature [12–14].

#### Model outputs

Model outputs were the total costs and QALYs gained. Total costs included all costs (medical, non-medical, indirect) and were summed across all model cycles to calculate the cost of illness burden of stroke among younger adults in Australia. Mean costs per case are also reported.

#### Sensitivity analysis

A series of one-way sensitivity analyses were conducted by varying input parameters to determine key drivers of the model. Ranges for each parameter were informed via the interquartile range from AVERT data where possible, with the exception of mRS probabilities at 12 months which were varied by 10%. Otherwise, the range was determined by assuming a null value for lower input parameters and by doubling the specified value for higher input parameters. The results from the one-way sensitivity analyses are presented in a form of a tornado diagram. Probabilistic sensitivity analyses were also conducted to examine uncertainty by incorporating the distribution of key variables (each parameter was sampled 5000 times).

### Loss of health-related quality of life

To gain an understanding of the loss of health-related quality of life due to stroke in younger adults, the estimated QALYs were compared with those of the age-matched general population sample, taking a lifetime horizon. First, a snapshot of the age at index stroke of all simulated patients was determined based on AIHW data. Age-dependent utility weights at 12-months post stroke calculated from the AQoL-4D [36] were then derived from AVERT (missing data: 5.25%) for the stroke population, whilst weights for the corresponding age in the general Australian population were based on Hawthorne et al. [37] (see Table 2). Remaining life expectancy or life years (LYs) of simulated patients with stroke for given ages were taken from the Markov microsimulation (i.e. model output in terms of the life years gained for each individual) and compared against those of the general population (i.e. the life table of general Australians) [21]. The differences in resulting QALYs (remaining LYs multiplied by utility for any given age group) between the populations were reported as the associated loss of health-related quality of life due to stroke.

**Table 2:** Utility scores for the Australian general population and the Australian AVERT stroke population

Age group (years)	Utility scores	
	General population [37]	AVERT stroke population (*n* = 926)
< 40	0.85	0.72 (*n* = 19)
40–49	0.81	0.65 (*n* = 47)
50–59	0.80	0.61 (*n* = 110)
60–69	0.80	0.56 (*n* = 215)
70–79	0.76	0.46 (*n* = 344)
80–85	0.70	0.35 (*n* = 191)

#### Sensitivity analysis

Age has inconsistently been found to be a predictor of health-related quality of life in stroke survivors over the long term [38–40]. In our base case analysis, it was assumed that utility remained constant. However, sensitivity analysis was conducted to account for changes in utility as the hypothetical population aged. In other words, utility was calculated using different age-dependent utility weights for the relevant age brackets over the cohort’s estimated lifespan (Table 2).

## Results

### Cost of illness

Based on the 13,555 cases of stroke among younger adults in Australia in 2018, the short term (5 years) cost of illness for younger people with stroke was estimated at AUD2.0 billion (USD1.6 billion) for 41,607 QALYs. This was equivalent to a mean 5 year cost per case of AUD149,180 [95%CI $37,805, $496,689] comprising $52,291 in medical costs, $18,180 in nonmedical costs and $78,709 for indirect costs, and 3.07 [95%CI 0, 4.25] QALYs per case (Table 3).

**Table 3:** Results for short term and long term modelled analyses

	Summed	Per case
**Short term – Base case**		
Effect (QALYs)	41,606.71	3.07
Cost (total), AUD	2,022,135,241	149,180
Medical	708,809,346	52,291
Nonmedical	246,423,317	18,180
Indirect	1,066,902,578	78,709
**Short term – Probabilistic sensitivity analysis**		
Effect (QALYs)	41,334.16	3.05
Cost (total), AUD	2,079,467,525	153,410
**Long term – Base case**		
Effect (QALYs)	200,972.80	14.83
Cost (total), AUD	3,385,762,610	249,780
Medical	1,351,299,747	99,690
Nonmedical	537,102,689	39,624
Indirect	1,497,360,174	110,466
**Long term – Probabilistic sensitivity analysis**		
Effect (QALYs)	192,775.16	14.22
Cost (total), AUD	3,707,241,600	273,496

QALYs = quality-adjusted life years; AUD = Australian dollar

Using a longer 30 year time horizon, the cost of illness for younger people with stroke was calculated as AUD3.4 billion (USD2.7 billion) for 200,973 QALYs. Per case, this equated to a mean 30 year cost of AUD249,780 [95%CI $60,814, $838,500] and 14.83 QALYs [95%CI 0, 25.50]. This cost comprised $99,690 in medical costs, $39,624 in non-medical costs and $110,466 for indirect costs (Table 3).

#### Sensitivity analysis

**Fig. 2:**
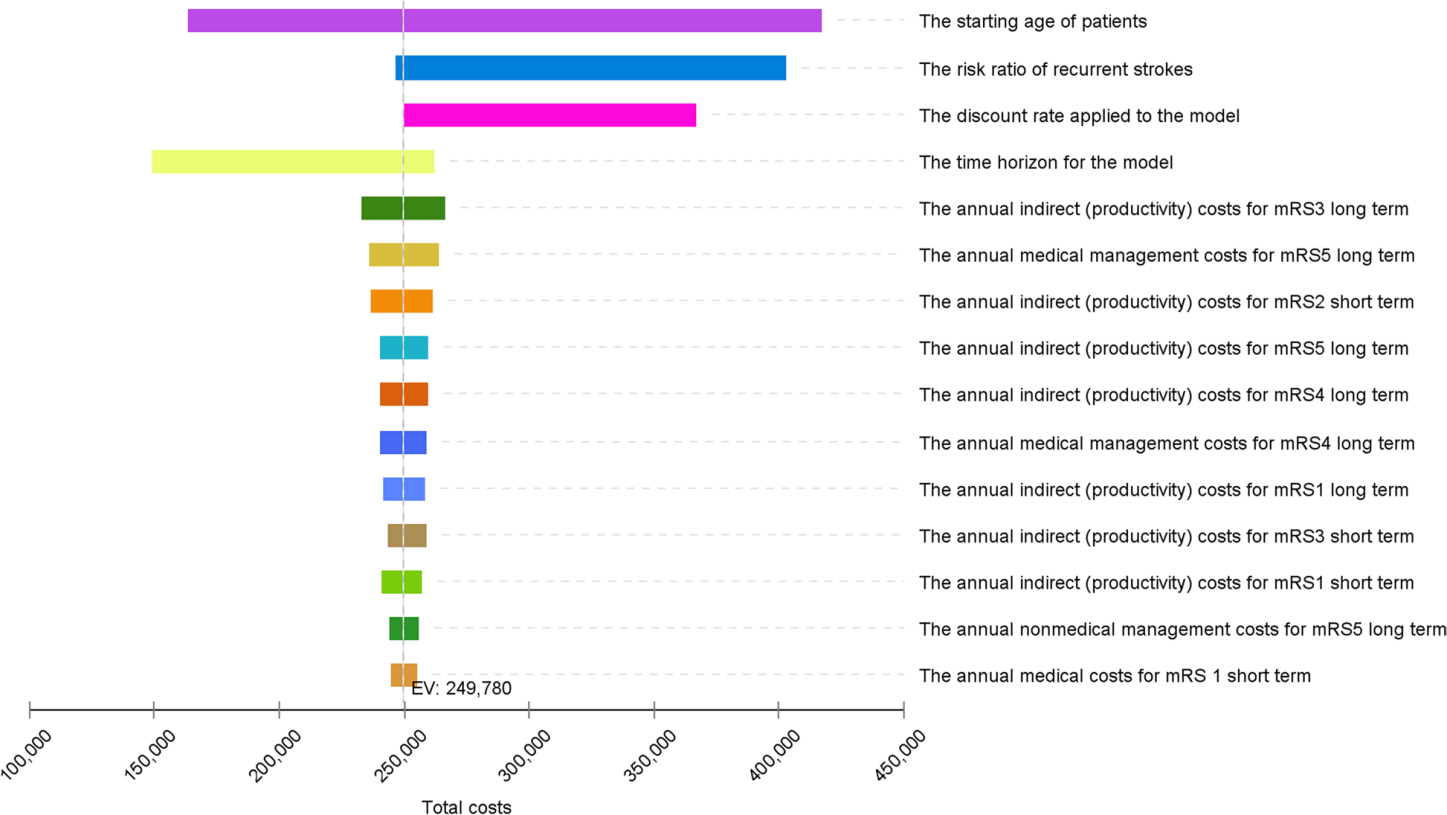
Tornado diagram. Notes. This diagram illustrates the change in the expected value (Australian dollars) when variables are tested within a range of input parameters

Overall, one-way sensitivity analyses found the results were most sensitive to the age of patients, the risk of recurrent stroke and the discount rate. Long term utility scores across all health states were found to be the least sensitive (Fig. 2).

**Fig. 3:**
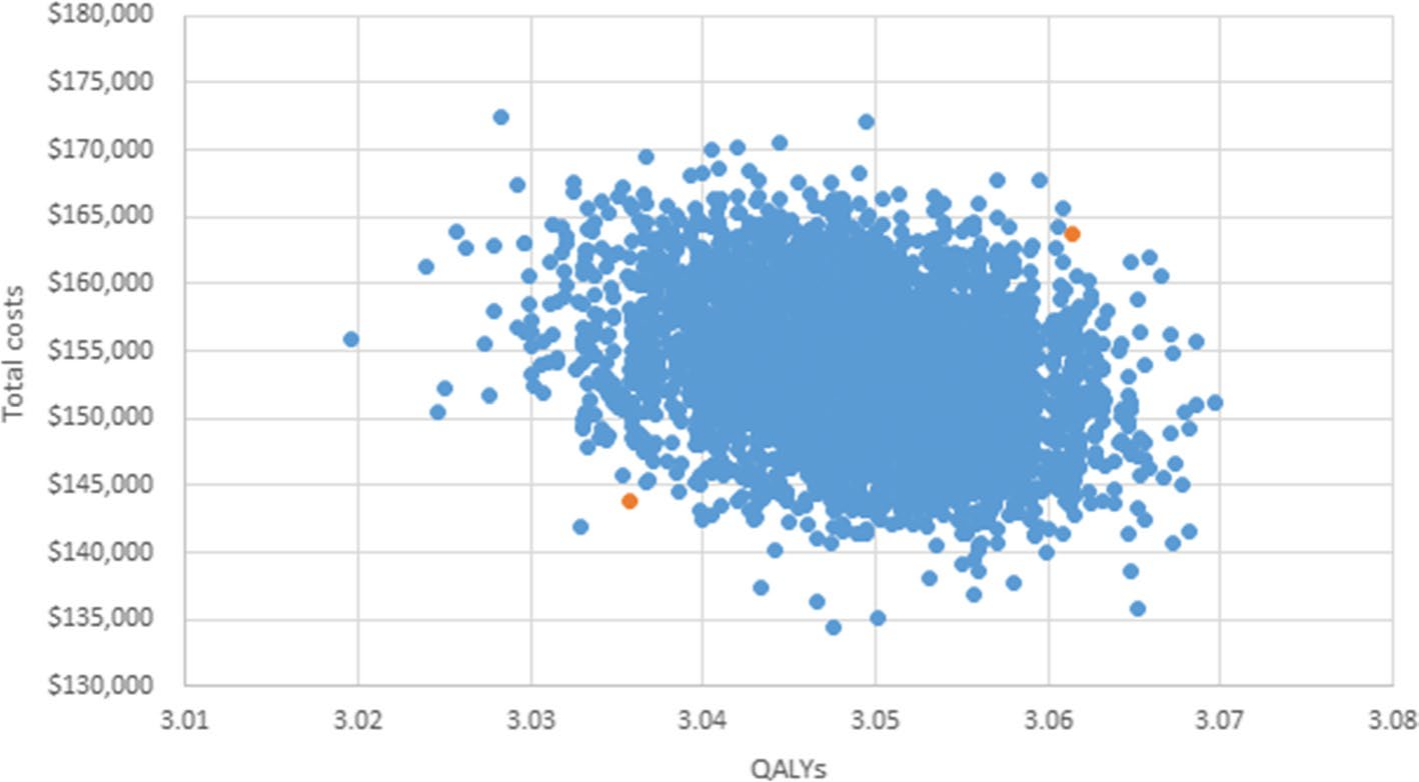
Results of probabilistic sensitivity analysis over the short term (5 years). Notes. This figure illustrates the range of outputs from the model for total costs (Australian dollars) and QALYs gained per case over 5 years. Orange colour denotes the 95% confidence interval. *QALYs* quality-adjusted life years

**Fig. 4:**
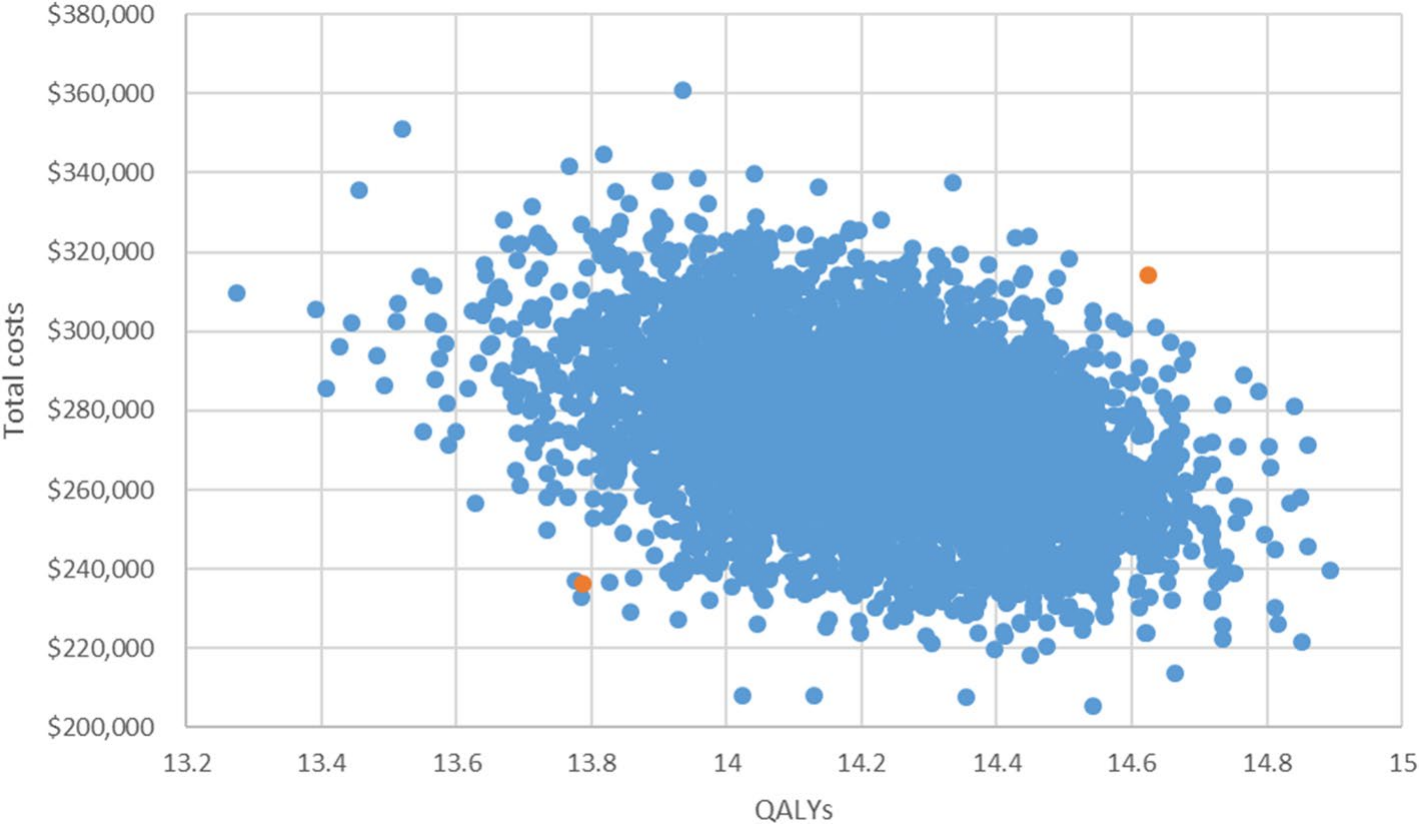
Results of probabilistic sensitivity analysis over the long term (30 years). Notes. This figure illustrates the range of outputs from the model for total costs (Australian dollars) and QALYs gained per case over the 30 years. Orange colour denotes the 95% confidence interval. *QALYs* quality-adjusted life years

Probabilistic sensitivity analysis results for the short term produced a mean cost per case of $153,410 [95%CI $143,834, $163,704] and mean QALYs of 3.05 [95%CI 3.04, 3.06] (Fig. 3). Probabilistic sensitivity analysis results for the long term produced a mean cost per case of $273,496 [95%CI $236,220, $314,368] and mean QALYs of 14.22 [95%CI 13.79, 14.62] (Fig. 4).

### Loss of health-related quality of life

As expected, the utility scores and remaining life expectancy derived from younger people with stroke were consistently lower than those elicited from the general population. Table 4 outlines the remaining LYs and loss of QALYs by age in the general population, compared to the stroke population. Overall, there was an average weighted loss of 4.58 LYs and 9.21 QALYs per younger adult with stroke.

**Table 4:** Results for loss of life years and loss of health-related quality of life

Age (years)	N	Remaining LYs		Loss of LYs	Loss of QALYs	
		General pop [21]	Stroke pop		Base case	Sensitivity analysis
< 25	499	61.90	52.15	9.75	15.37	16.76
25–34	657	53.15	45.18	7.97	12.91	14.92
35–44	1649	43.53	37.35	6.18	10.98	12.58
45–54	3842	34.15	29.41	4.74	9.42	10.47
55–64	6908	25.19	21.78	3.41	7.88	8.38
Average weighted loss				4.58	9.21	10.11

Notes. The health burden of stroke compared to that of the general population of the same age for the estimated 13,555 hypothetical younger adult stroke cases in 2018. *LYs* life years, *QALYs* quality-adjusted life years, *pop* population

#### Sensitivity analysis

Sensitivity analysis considering the association of decreased utility with increased age produced greater loss of QALYs at all levels. This is due to greater differences in age-matched utilities between the stroke and the general population (Table 2). The sensitivity analysis showed an average weighted loss of 10.11 QALYs per case.

## Discussion

### Cost of illness

This study sought to estimate the burden of stroke among younger adults (18–64 years) in Australia. As with all models, the validity of a model is largely dependent on model inputs and assumptions applied. A key strength of this study was the use of Australian trial data to inform the model, as opposed to sole reliance on literature.

Previous cost of illness studies for stroke in Australia include the North East Melbourne Stroke Incidence Study (NEMESIS), which examined the incidence of stroke in a community setting between 1996 and 1997. The reported lifetime societal cost of first-ever stroke in 1997 was AUD1.3 billion, resulting in an average cost of $44,428 [41]. Latest estimates from NEMESIS, updated using 10-year longitudinal data, reported a burden of $3.1 billion (based on an estimated 25,351 first-ever ischemic strokes and 5356 first-ever intracerebral haemorrhages in 2010) or lifetime costs of $103,566 for ischemic stroke and $82,764 for intracerebral haemorrhage (reference year: 2010 AUD) [6]. In light of these previous cost estimates, it is perhaps somewhat surprising that we estimate the lifetime burden of stroke among younger adults in Australia, to be AUD3.4 billion or $249,780 per case.

Firstly, our cost of illness study has focussed on the working age population and it is expected that the indirect costs of productivity losses for our population will be substantially higher than for an unselected population with a mean age of 73 in NEMESIS [41]. Secondly, there are important differences between the populations of working aged people included in these two studies. In NEMESIS, only 45% of those of working age were working prior to their stroke whereas this was 67% of participants in the AVERT. Thirdly, the methods used to estimate productively losses are very different. In our analysis, the human capital method was used [42] with approximately 53% of costs in the short term and 44% in the long term attributable to productivity losses. The NEMESIS investigators [41] used the frictional cost method and indirect costs only accounted for 6% of total first year costs in that study. The human capital approach includes any hour of potential working life not worked due to illness whereas the frictional method only includes productivity loss during the period it takes to fully replace a worker, a much shorter time.

The NEMESIS studies were performed using an incidence-based approach to measure the cost of illness (similar to our study). The number of new cases in a year were modelled over the specified time horizon, and present and future costs were valued in accordance for a given reference year. On the other hand, prevalence-based studies measure costs of all new and existing cases to give a snapshot of costs for a given reference year. This is the approach employed in the Deloitte Access Economics study, in which the economic costs of stroke were reported as $5 billion in 2013, with $3 billion due to lost productivity alone (reference year: 2012 AUD) [4], and more recently updated with 2020 estimates of $6.2 billion (with productivity losses accounting for 3.6 billion) (reference year: 2020 AUD) [43]. Interestingly the Deloitte study accounted for other variables not considered in our model such as presenteeism (where a worker is at work but is not fully productive directly as a result of their disease) and transfer costs [4, 43]. Therefore, the results of this study are not directly comparable to our study.

### Loss of health-related quality of life

With regard to the associated loss of health-related quality of life, the results from our analysis were slightly higher than those of a previous study that used NEMESIS data and the AQoL tool. In this study, Cadilhac et al. reported an approximate loss of 7.24 QALYs and 8.88 QALYs for ischemic stroke and intracerebral haemorrhage respectively, across the population (including persons over 65 years) [44]. It is somewhat surprising that our result of 9.21 QALYs was not larger in comparison due to the greater loss of life years experienced by younger patients. The differences in results may be explained by differences in the case fatality rate used – in our study this was applied at 1-year (8.66% specific to patients under 65 [9]), followed by a lifetime risk of death from recurrent stroke. In Cadilhac et al’s study, stroke-related mortality based on NEMESIS data was calculated up to 10 years post stroke by gender and age group, however the specific rate was not reported (overall 1-year case fatality indicated at 37% [45]).

### Implications

Our findings shed light on the costly burden of stroke among younger adults, specific to Australia. Indirect costs are particularly high in this population compared with older adults, stemming from absence and/or premature exit from the labour force as a result of stroke.

Serious gaps in rehabilitation also impede recovery and return to work. A 2016 audit in Australia found that none of the 121 rehabilitation services audited met all 10 essential elements of the National Rehabilitation Stroke Services Framework and a majority of patients were not adequately supported in the transition back to life [46]. A recent analysis of global AVERT data found that 25% of younger stroke survivors did not receive rehabilitation upon discharge from hospital, and this was even more pronounced in patients aged between 18 and 45 years [47]. Additionally, younger stroke survivors were more likely than their older counterparts to experience unmet needs across the various domains of life (health, everyday living, leisure activities, support and finances) [48]. For those working prior to their stroke, three in every four experienced a change in work activities, and 60% of those requiring help to return to work reported receiving inadequate assistance [48].

Research suggests that 80–90% of strokes are preventable [49]. Modifiable key risk factors such as dyslipidemia influenced by unhealthy behaviours are increasingly prevalent among young people in Australia. For example, the prevalence of overweight and obesity in persons aged 18–24 years increased from 39% in 2015 to 46% in 2017 [50]. The growth in risk factor prevalence is consistent with global trends that stroke amongst the working age population is on the rise [51]. With the incidence of stroke among younger adults expected to rise [51], this information may be used to assist with policy formulation, resource allocation, and priority setting to better support younger stroke patients. The results may also inform evaluations of stroke interventions in Australia targeting persons under 65, by quantifying their return on investment.

### Limitations

Our study has several limitations. Our analysis was based on a selected population and may not be fully representative of the younger adult stroke population in Australia. While we tried to consider this through the use of AIHW data (patient characteristics and age-dependent mortality due to recurrent stroke), this may not fully account for the inherent differences between the trial population which was subjected to AVERT inclusion and exclusion criteria, and the general stroke population. For example, AVERT excluded patients with clinically significant pre-morbid levels of disability (mRS score > 2) who may be less likely to be working, and patients who experienced early deterioration. The trial data used were also subject to recall bias during face-to-face collection of data at 3 and 12 month time points, and most data were self-reported (e.g. equipment and aids; informal care; employment), though all hospital and rehabilitation data were obtained via patient level health records. Further, as costs were sourced from the literature, these may be inaccurate for some of the smaller, more variable costs – for example, the costs of equipment.

In terms of our analysis in estimating the loss of health-related quality of life, the difference in utility scores of the stroke population in our study to those in other studies, was noted. The utility scores used in our study were lower than in other studies [2, 44], meaning greater net differences in utilities compared to the age-matched general population. Estimates of utility can vary and are influenced by elicitation methods [52, 53]. Lastly, the age-dependent utilities applied in the calculation of loss of health-related quality of life were those elicited at 1 year post stroke. It could be argued that participants may become accustomed to a lower health state and therefore experience improved utility in the longer term. For example, one study reported an increase in the utility of severe stroke survivors from 0.38 to 0.45, between 6 months to 2 years post stroke [39]. Conversely, participants may also experience disutility over time, although there is also evidence that utility may remain stable [39].

## Conclusions

To the best of our knowledge, this is the first study quantifying the cost of illness and associated loss of health-related quality of life specific to stroke among younger adults in Australia. This study highlights the disproportionate burden of stroke in adults aged less than 65 years. The results of our study should be used to inform policy focusing on empowering individuals to take charge of their health; raising the awareness of stroke among younger adults in the public, and in acute settings; and increasing support for rehabilitation and the transition back to life after stroke.

## Supplementary Information

**Additional file 1.**

## Data Availability

The datasets used and/or analysed during the current study are available from the corresponding author on reasonable request.

## Abbreviations

AIHW: Australian Institute of Health and Welfare
AQoL: Assessment of Quality of Life
AUD: Australian dollars
AVERT: A Very Early Rehabilitation Trial
GP: General practitioner
IHPA: Independent Hospital Pricing Authority
LYs: Life years
mRS: modified Rankin Scale
NEMESIS: North East Melbourne Stroke Incidence Study
QALYs: Quality-adjusted life years
QHES: Quality of Health Economic Studies
USD: US dollars
