# Correction to: The economic and health burden of stroke among younger adults in Australia from a societal perspective

**DOI:** 10.1186/s12889-022-12712-0

**Published:** 2022-02-23

**Authors:** Elise Tan, Lan Gao, Janice M. Collier, Fiona Ellery, Helen M. Dewey, Julie Bernhardt, The AVERT Trial Collaboration Group, Marj Moodie

**Affiliations:** 1 Deakin Health Economics, Institute for Health Transformation, Deakin University, Geelong, Australia; 2 Florey Institute of Neuroscience and Mental Health, University of Melbourne, Heidelberg, Australia; 3 Eastern Health Clinical School, Monash University, Box Hill, Australia

**Correction to: BMC Public Health 22, 218 (2022)**

**https://doi.org/10.1186/s12889-021-12400-5**

Following publication of the original article [1], the authors identified an error in Fig. 1. The correct figure is given below.

**Fig. 1:**
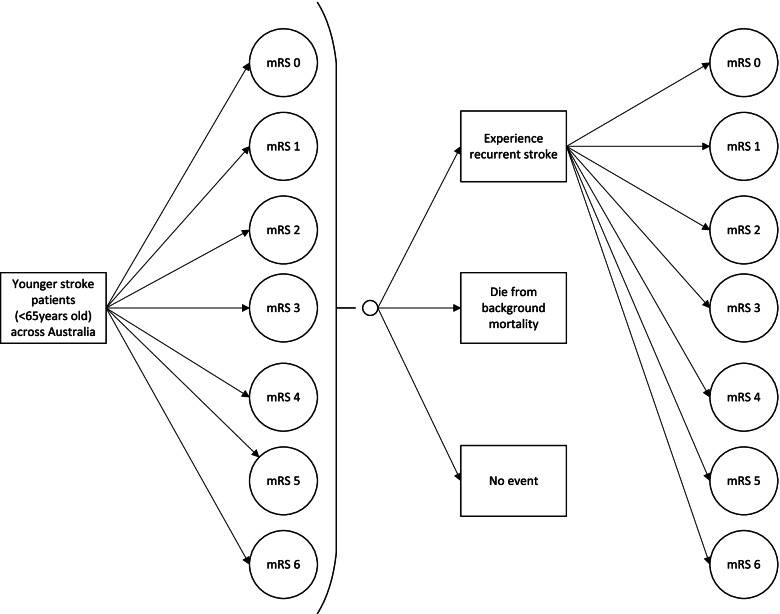
Structure of the cost of illness microsimulation model. Notes. The assumption is that a patient’s health state cannot improve. *mRS* modified Rankin Scale

The original article [1] has been corrected.
