# Supplementary material for: The economic and health burden of stroke among younger adults in Australia from a societal perspective
Source: BMC Public Health. 2022 Feb 3;22:218. doi: 10.1186/s12889-021-12400-5 (PMC8811989; doi:10.1186/s12889-021-12400-5)
Supplement: Supplementary file 1 — Additional file 1. [file 12889_2021_12400_MOESM1_ESM.docx]

# **Supplementary: Members of the AVERT Trial Collaboration Group**

## AVERT committees, advisors and coordinating centres

**Management Committee**

Julie Bernhardt^2^ (Chair), Leonid Churilov^2^, Janice Collier^2^, Helen Dewey^3,4^, Geoffrey Donnan^2^, Fiona Ellery^2^, Peter Langhorne^5^, Richard Lindley^6^, Marjory Moodie^1^, Brooke Parsons^24^ (Consumer Representative), Amanda Thrift^7^.

**Trial Steering Committee**

Geoffrey Donnan^2^ (Co-Chair), Helen Dewey^3,4^ (Co-Chair), Julie Bernhardt^2^, Peter Langhorne^5^, Marjory Moodie^1^, Brooke Parsons^24^ (Consumer Representative) and Main Investigators (MIs) from all participating hospitals.

**International Advisors**

Bent Indredavik^8^, Torunn Askim^8^.

**Data Monitoring Committee**

Phillip Bath^9^ (Chair), Christopher Bladin^10^, Christopher Reid^7^, Stephen Read^11^, Cathy Said^12^.

**Outcomes Committee**

Sandy Middleton^13^ (Chair), Judith Frayne^14^, Velandai Srikanth^15^.

**Country Leaders and Grant Holders**

Australia: Julie Bernhardt^2^, (NHMRC: Helen Dewey^3,4^, Julie Bernhardt^2^, Geoffrey Donnan^2^, Amanda Thrift^7^, Robert Carter^1^, Richard Lindley^6^) (NHMRC: Julie Bernhardt^2^, Geoff Donnan^2^, Richard Lindley^6^, Amanda Thrift^7^, Peter Langhorne^5^, Marjory Moodie^1^, Helen Dewey^3,4^, Leonid Churilov^2^).

United Kingdom: Peter Langhorne^5^, (CHSS: Peter Langhorne^5^, Olivia Wu^5^, Julie Bernhardt^2^, Matthew Walters^5^, Claire Ritchie^16^, Lorraine Smith^5^), (TSA: Peter Langhorne^5^, Olivia Wu^5^, Anne Ashburn^17^, Helen Rodgers^18^, Julie Bernhardt^2^), (HTA: Peter Langhorne^5^, Anne Ashburn^17^, Julie Bernhardt^2^, Helen Rogers^18^, Olivia Wu^5^).

Northern Ireland: Sheila Lennon^19^, (NICHS: Sheila Lennon^19^, Michael Power^20^, Julie Bernhardt^2^).

Singapore: Shahul Hameed^21^, (Singhealth: Shahul Hameed^21^, Ratnagopal Pavanni^21^, Peter Lim^21^, Julie Bernhardt^2^, Dawn Tan^21^).

**Statistics and Data Management**

Leonid Churilov^2^, Tim Brewer^2^, Janice Collier^2^, Nick Haritos^2^, Edwin Leong^2^, Cecilia Li^2^, Caesar NayWin^2^, Marcus Nicol^2^, Liudmyla Olenka^2^, Li Chun Quang^2^.

**Health Economics**

Marjory Moodie^1^, Robert Carter^1^, Silvia Hope^1^, Lauren Sheppard^1^, Kiusiang Tay- Teo^1^, Olivia Wu^5^.

**Cognition**

Toby Cumming^2^, Thomas Linden^22^.

**Trial Coordinating Centres**

**The Florey Institute of Neuroscience and Mental Health, Melbourne, Australia.** Karen Borschmann^2^, Jan Chamberlain^2^, Janice Collier^2^, Toby Cumming^2^, Fiona Ellery^2^, Teresa Occhiodoro^2^, Helen Palfreeman^2^, Tara Purvis^2^, Bernadette Sirgo^2^, Nick Tiliacos^2^, John Van Holsteyn^2^, Henry Zhao^2^.

**University of Glasgow, Glasgow, United Kingdom.** Beverly Armstrong^5^, Louise Craig^5^, Fiona Graham^5^, Lynn Legg^5^, Rosemary Morrison^5^, Heather Moorhead^5^, Lorraine O’Donohue^5^, Susan Rogers^5^, Myra Smith^5^.

**University of Central Lancashire, Preston, United Kingdom.** Denise Forshaw^23^, Jane Fitzgerald^23^.

## List of AVERT collaborators by country

Main Investigators are listed first (MI).

**Australia**

E Hibbert^4,25^, R Melling^4^, S Petrolo^4^, T Purvis^2,4^, H Williamson^4^, J Ancliffe^26^, K Clarke^25^, H Maccanti^25^, L Marr^25^, S Plumb^25^, J Quiney^25^, L Werner^25^, M Baxter^27^, M Davis^27^, L Sundararajan^27^, R Chen^28^, K Bainbridge^29^, B Killey^29^, R Sheedy^29^, K Richardson^14^, N Crawshaw^30^, J Luker^30^, C Wood^30^, S Choat^30^, L Mackey^31^, T Wijeratne^31^, M Shannon^32^, R Gerraty^32^, N Austin^33^, S Pomfret^33^, M Tinsley^33^, V Crosby^34^, R Grimley^35^, D Rowley^35^, T Beckwith^36^, L Cormack^36^, P Groot^37^, S Smith^38^, V Bramah^39^, R Errey^39^, M Halpin^39^, V Molan^39^, D Wheelwright^39^, N Wilson^39^, W Zhang^39^, G Auld^40^, J Cramb^41^, A Moore^42^, A Robertson^42^, L Tighe^43^, O Katalinic^44^, M Spear^44^, S Cox^45^, C Tse^45^, P Andersen^46^ (MIs), P Adams^4^, L Augoustakis^4^, S Batcheler^4^, S Berney^4^, V Cobani^4^, B Cohen^4^, H Dewey^3,4^, S Gangi^4^, N Giofre^4^, C Gordon^4^, L Hegarty^4^, M Hindson^4^, F Horvath^4^, S Kalinowski^4^, A Kleine^4^, S Kramer^4,25^, J Lawrence^4^, S Lindquist^4^, N Logan^4^, A Macdonell^4^, J Matlioski^4^, N McDonough^4^, S McLennan^4^, M McNamee^4^, L Miller^4^, C Nall^4^, E Nelson^4^, K Ng^4^, Z Nicholas^4^, C Nunn^4^, K Owen^4^, E Plant^4^, L Proud^4^, D Quah^4,25^, K Rodway^4^, S Sertori^4^, V Sheldon^4^, L Sherry^4^, S Speare^4,25^, K Stansfeld^4^, N Studden^4^, Z Teoh^4^, L Twist^4^, G Velupillai^4^, L Walker^4^, K Wall^4^, A Warwick^4^, R Wharrie^4^, J Wilson^4^, H Worboys^4^, D Young^4^, M Bryant^26^, B Doran^26^, M Field^26^, P Fogliani^26^, A Haber^26^, G Hankey^26^, D Hendrie^26^, V Jackaman^26^, A Jacobsen^26^, S Jose^26^, R Lim^26^, R Louis^26^, S Nanthakumar^26^, S Pain^26^, A Power^26^, B Rappeport^26^, J Reynolds^26^, L Smith^26^, S Tombe^26^, A Wesseldine^26^, T West^26^, E Abeykoon^25^, W Apirutvorrachod^25^, L Attard^25^, S Behanan^25^, D Brown^25^, K Buchanan^25^, D Butler^25^, M Camac^25^, S Davis^25^, D Diocera^25^, N Gan^25^, C Gendre^25^, J Germaine^25^, P Hand^25^, L Maurenbrecher^25^, J McCulloch^25^, S Mcritchie^25^, M Ong^25^, R Pachett^25^, L Pesavento^25^, H Power^25^, R Reilly^25^, M Sawers^25^, G Silva^25^, C Stevens^25^, L Taylor^25^, T Timms^25^, M Ugalde^25^, A Vardy^25^, J Wallace^25^, S Walsh^25^, E Whatley^25^, E Winter^25^, E Butler^27^, K Caspers^27^, E Coulter^27^, S Shaw^27^, F Kent^27^, H Lack^27^, F Leavold^27^, J Lord^27^, J Martin-Francisco^27^, R Mohanraj^27^, R Nelson^27^, T O'Neill^27^, R Otto^27^, J Parker^27^, V Rees^27^, B Stevens^27^, RI Lindley^28^, J Bindra^28^, R Dongre^28^, N Downey^28^, M Ferris^28^, L Gibson^28^, R Gonzalez^28^, M Kinniburgh^28^, M Lazaridou^28^, D McCormack^28^, R Singh^28^, A Stepney^28^, Y Tria^28^, O Aitchison^29^, L Bray^29^, K Clatworthy^29^, S Coghill^29^, M Collins^29^, L Cornwall^29^, J Dow^29^, P Gates^29^, S Gillett^29^, N Johnson^29^, S Joseph^29^, K Kopelke^29^, R Lam^29^, R Levy^29^, N Lloyd^29^, S Logan^29^, G McPherson^29^, M Newth^29^, C Parsons^29^, K Powles^29^, M Rebis^29^, T Samakowidic^29^, L Sanders^29^, S Savickas^29^, J Shrimpton^29^, H Smith^29^, L Smith^29^, J Spehr^29^, J Summers^29^, G Taylor^29^, M Thackeray^29^, B Wilkinson^29^, J Frayne^14^, E Barber^14^, L Bode^14^, A Brakey^14^, K Chand^14^, P Christin^14^, G Crook^14^, D Delrosario-Kelly^14^, R Descallar^14^, A Deutsch^14^, S Easo^14^, M Farquhar^14^, P Fergus^14^, J Ford^14^, E Hamson^14^, M Hlaing^14^, E Hope^14^, J Lacivita^14^, J Laurenson^14^, K Lock^14^, N Ly^14^, K McKay^14^, C Mill^14^, K Moloney^14^, L Price^14^, T Terry^14^, A Tyers^14^, S Willems^14^, R Woolstencroft^14^, C Archer^30^, D Benham^30^, M Billinger^30^, M Bronca^30^, S Curchin^30^, C Dickie^30^, M Dixon^30^, D Douglass^30^, M Enomoto^30^, K Ernst^30^, L Fries^30^, S George^30^, E Green^30^, L Hamilton^30^, Z Harris^30^, T Heard^30^, G Hunt^30^, N Jamieson^30^, M Mackenzie^30^, H McKearney^30^, B Oermann^30^, C O'Reilly^30^, T Pearson^30^, N Reid^30^, L Rodda^30^, D Scutcheon^30^, C Simons^30^, R Smith^30^, L Tait^30^, J Troake^30^, D Usher^30^, C Abela^31^, S Ashoka^31^, C Chen^31^, T Cheng^31^, V Chong^31^, S Cooke^31^, A Fok^31^, L Galang^31^, C Grant^31^, S Karageorge^31^, K Kat^31^, L Keo^31^, B Lee^31^, A Luscombe^31^, J Mackay^31^, M Minett^31^, J Mizen^31^, P Nim^31^, N Nunlist^31^, V Patel^31^, M Pathirage^31^, A Paton^31^, M Pombuena^31^, N Rathnayake^31^, L Rhodes^31^, M Sequeira^31^, S Smart^31^, S Somaratne^31^, N Sta Maria^31^, L Talbot^31^, R Tecle^31^, S Allen^32^, R Boyle^32^, N Fatchen^32^, N Hendley^32^, A Hyde^32^, M Inal^32^, P Kalubowilage^32^, M Laverde^32^, K Lawless^32^, A McFadyen^32^, K Peters^32^, C Pugh^32^, C Qin^32^, J Robertson^32^, S Smee^32^, R Tomlinson^32^, V Wang^32^, F Williams^32^, D Woolley^32^, R Yawieriin^32^, L Allport^33^, C Ang^33^, L Armitage^33^, E Blundell^33^, A Courtney^33^, M Dela Costa^33^, T Devi Thapa^33^, P Diwakar^33^, M Dulleh^33^, J Francis^33^, P Cic^33^, G Gellie^33^, C Gill^33^, D James^33^, S Lee^33^, T Mai^33^, K Majcher^33^, C Mawson^33^, G Newton^33^, N Qiu^33^, E Ragonton^33^, L Roberts^33^, H Saitamis^33^, L Stanwell^33^, L Ting^33^, P Xu^33^, L Yin^33^, K Broadhead^34^, J Church^34^, R Collins^34^, K Everitt^34^, M Fisher^34^, K Hochmuth^34^, N Jones^34^, A Lieschke^34^, E McCarthy^34^, C McGlone^34^, D Morey^34^, D Neilson^34^, S Spry^34^, M Vile^34^, I Rosbergen^35^, E Ahern^35^, L Anderson^35^, J Boreham^35^, R Devin^35^, R Doolan^35^, M Dyke^35^, L Griffiths^35^, K Guest^35^, D Hecita^35^, N Kendal^35^, J Koltermann^35^, M Lacy^35^, S Lebeter^35^, D Lloyd^35^, M Matthews^35^, C McAuley^35^, A Pollock^35^, M Pyke^35^, T Rogers^35^, S Street^35^, G Styles^35^, A Tampiyappa^35^, J Trinder^35^, T Verral^35^, K Walker^35^, C White^35^, J Arriagada^36^, C Babenschneider^36^, D Blacker^36^, S Bennett^36^, S Connor^36^, J Cowmeadow^36^, N Daniel^36^, G Edmonds^36^, M Faulkner^36^, M Garcia-Vega^36^, K Kruger^36^, B Martial^36^, P McGinley^36^, H Mountford^36^, V Riley^36^, N Smith^36^, F Stepan^36^, S Tilley^36^, S Whisson^36^, J Bailey^37^, K Ballinger^37^, C Bell^37^, B Camilleri^37^, C Charnley^37^, D Crabbe^37^, S Crossland^37^, N Edirimanna^37^, C Fitzgerald^37^, C Gibbins^37^, J Gibbs^37^, K Hirst^37^, A Kennedy^37^, E Klose^37^, K McDowall^37^, S Miller^37^, R Morgan^37^, A Noonan^37^, M North^37^, M Oliver^37^, K Richards^37^, T Russell^37^, N Scott^37^, A Shlanski^37^, A Traynor^37^, R Adams^38^, C Banks^38^, K Burke^38^, S Hewat^38^, B McKenna^38^, M McKimmie^38^, L Polmear^38^, M Traumanis^38^, S Whiteman^38^, M Bakshi^39^, S Bracher^39^, M Bryant^39^, W Byrnes^39^, T Denton^39^, N DeVries^39^, P Fay^39^, P Galbraith^39^, T Gallaher^39^, O Haidar^39^, K Holgate^39^, K Hozack^39^, N Jackson^39^, S Kipps^39^, S Lerner^39^, R Markus^39^, R Merheb^39^, C Naismith^39^, G Nolan^39^, R Odelli^39^, K Page^39^, P Sangvatanakul^39^, T Simpson^39^, P van Vliet^39^, K Walch^39^, S Walker^39^, T Yasue^39^, R Baker^40^, K Cousins^40^, M Fairbrother^40^, K Hutchinson^40,46^, M Maclean^40^, E Maher^40^, D Mills^40^, S Ohlback^40^, J Sturm^40^, M Tooth^40^, J Watkins^40^, P Atkinson^41^, J Conrad^41^, D Fichera^41^, S Follent^41^, C Gilbert^41^, M Herzig^41^, S Kohler^41^, S McCracken^41^, L Nunan^41^, S Roberts^41^, J Shelley^41^, S Varendorff^41^, A Wills^41^, J Britton^42^, A Burgess^42^, T Coates^42^, J Croft^42^, E Greening^42^, J Holland^42^, P O'Brien^42^, R Strong^42^, S Bilston^43^, J Black^43^, K De Rivera^43^, I Dwyer^43^, S Gissane^43^, K Heckenberg^43^, S Jackson^43^, A Maclagan^43^, L O'Hare^43^, H Patel^43^, J Pearce^43^, C Scanlan^43^, K Seymour^43^, M Symington^43^, A Tyers^43^, A Waite^43^, K Wiesner^43^, P Brown^44^, E Difuntorum^44^, S Gilbert^44^, J Henderson^44^, D James^44^, H Janssen^44^, E Lane^44^, S Lowndes^44^, D Smith^44^, S Thompson^44^, D Weaver^44^, S Weston^44^, S Wright^44^, J Adrian^45^, M Doughty^45^, J Kok^45^, R McGrath^45^, T Morris^45^, A Pickup^45^, E Ray^45^, R Richardson^45^, M Sims^45^, C Thompson^45^, K Trinh^45^, N Walton^45^, F Whittaker^45^, J Burrows^46^, M Dawson^46^, D Griffiths^46^, G Harris^46^, P Kavalieros^46^, B O'Brien^46^, K Roberts^46^, J Watkins^46^, C Whyatt^46^.

**New Zealand**

A McRae^47^, G Wavish^47^ (MIs), F Anos^47^, J Armstrong^47^, E Au^47^, A Barber^47^, C Bates^47^, M Bertulfo^47^, A Boggs^47^, F Burgess^47^, K Cassels-Brown^47^, M Chiu^47^, S Dass^47^, N Duff^47^, J Farrell^47^, W Foster^47^, D Fuertez^47^, C Gadhvi^47^, S George^47^, A Green^47^, L Harvey-Fitzgerald^47^, L Hau^47^, L Hayward^47^, D Holman^47^, K Huggins^47^, M Jacobs^47^, A John^47^, H Kaur^47^, T Lagerstedt^47^, J Lee^47^, R Llenes^47^, L Lyons^47^, S Magandi^47^, M Martin^47^, S Mathew^47^, T Mathew^47^, D McKellar^47^, E Moss^47^, KL Nand^47^, K Nicol^47^, F Peterson^47^, A Prasad^47^, K Quick^47^, E Revell^47^, S Roy^47^, J Ryan^47^, N Samadi^47^, B Scrivener^47^, J Slow^47^, S Tharakan^47^, J Torrens^47^, E van Bysterveldt^47^, C Villaluz^47^, S Yang^47^.

**Malaysia**

Katijjahbe MA^48^ (MI), Ai Sing G^48^, Azlina A^48^, Azmi MI^48^, Efri MH^48^, Fadilah AZ^48^, Fathuddeen H^48^, Haryani H^48^, H Hussien^48^, Izuani M^48^, ZC Man^48^, Man Ying C^48^, Mashitoh KM^48^, Noor Azah A^48^, Norlinah MI^48^, Norliza I^48^, Ravinder KB^48^, Rohaizah R^48^, Rosnita K^48^, Rozita A^48^, Safwan J^48^, R Sahathevan^48^, Shahrul I^48^, Sharifah SM^48^, Tan HJ^48^, Wan Nafisah WY^48^, YL Yee^48^, Zaharah MA^48^, Zunaidah AS^48^.

**Singapore**

D Tan^21^, MT Ahmad^21^, S Hameed^21^ (MIs), MFB Bakari^21^, J Britto^21^, JJ Chen^21^, S Choo^21^, M Faizal^21^, FK Fong^21^, S Hong^21^, J Ja'afar^21^, Z Ke^21^, G Koh^21^, CK Lee^21^, YF Lee^21^, P Lim^21^, GM Lim^21^, SH Ninhadi^21^, G Ong^21^, T Pei Pei^21^, V Penero^21^, N Rahim^21^, P Ratnagopal^21^, K Saleh^21^, HC Seow^21^, E Sim^21^, CK Tan^21^, PY Tay^21^, I Teo^21^, S Thilarajah^21^, PHJ Wong^21^, WP Wong^21^, S Yeap^21^.

**United Kingdom**

M Macleod^49^, D Neal^50^, J Coyle^51^, M Keeling^51^, S Louw^52^, J Furnace^53^, MJ Macleod^53^, C Gordon^54^, J Kwan^54^, L Redpath^54^, K Saunders^54^, R Howes^55^, A Lacey^55^, P Meakin^55^, E Feely^56^, S Kirk^56^, M Barber^57^, D Esson^57^, B Wroath^20^, C Charnley^58^, M James^58^, G Gunathilagan^59^, J Sampson^59^, G Thomas^59^, P Browne^60^, C McGoldrick^60^, D Mullan^60^, C Price^61^, V Riddell^61^, V Green^62^, K Mitchelson^63^, L Mokoena^63^, R McGeown^64^, S Tauro^64^, S Appleby^65^, S Brotheridge^65^, M Prescott^65^, E Hakim^66^, J Herman^66^, T Norman^66^, K Buck^67^, M Walters^16^, H Hunter^68^, A Nair^69^, G Cloud^70^, J Hunt^71^, R Latif^71^, S Burgess^72^, T Elder-Gracie^72^, K Robinson^73^, K Mason^74^, C Douglas^75^ (MIs), A Anderson^49^, K Armstrong^49^, K Baird^49^, D Balfour^49^, M Boyd^49^, J Cameron^49^, C Carswell^49^, C Clanachan^49^, L Cuthill^49^, I Devoy^49^, S Forsyth^49^, J Gavin^49^, M Hughes^49^, E Marr^49^, S McAuley^49^, E McCagherty^49^, K McCallum^49^, N McDonald^49^, C McGhee^49^, TA McIntyre^49^, L Noonan^49^, A Smart^49^, R Walshe^49^, J Allison^50^, G Ball^50^, S Board^50^, H Brunt^50^, C Buckley^50^, C Carroll^50^, D Hayward^50^, T Hutchinson^50^, E Jones^50^, E Keeling^50^, E Marsh^50^, N Mead^50^, H Smith^50^, C Vickers^50^, B Williams-Yesson^50^, D Wood^50^, L Ackroyd^51^, C Brown^51^, K Donnan^51^, N Dyer^51^, H Green^51^, G Kilbride^51^, C Nicholson^51^, M Porteous^51^, A Annamalai^52^, A Barkat^52^, S Crawford^52^, M Fawcett^52^, D Harvey^52^, V Hogg^52^, A Hughes^52^, J Kemp^52^, J Morrison^52^, K Storey^52^, T Thompson^52^, J Bell^53^, K Bennett^53^, M Bruce^53^, R Clarke^53^, H Cowie^53^, H Gow^53^, J Irvine^53^, A Joyson^53^, S MacDonald^53^, A Macvicar^53^, N Murphy^53^, J Robertson^53^, J Bell^54^, R Burrow^54^, C Clarke^54^, C Dickson^54^, G Hann^54^, M Heath^54^, S Heath^54^, A Hewett^54^, R Humphrey^54^, B Longland^54^, A Orpen^54^, C Ovington^54^, J Page^54^, E Rogers^54^, K Toombs^54^**,** D Ames^55^, S Banerjee^55^, E Beranova^55^, S Berry^55^, MJ Burke^55^, V Cassama^55^, K Collins^55^, J Crow^55^, A Dunne^55^, C Gomez^55^, A Hawkins^55^, K Hellier^55^, SA Howard^55^, A Kar^55^, E Lambert^55^, H Lee^55^, C Mandri^55^, J Moye^55^, E Murtagh^55^, J Pushpa-Rajah^55^, J Richardson^55^, T Sachs^55^, J Stilwell^55^, V Tilley^55^, P Wilding^55^, N Wilson^55^, P Cassidy^56^, A Chalmers^56^, C Duguid^56^, N Hughes^56^, J Hutton^56^, K Lapsley^56^, J Lee^56^, A Murray^56^, L Weir^56^, M Whitelaw^56^, H Armit^57^, C Devlin^57^, R Duncan^57^, C Forman^57^, K Frame^57^, L Hogg^57^, P McLeod^57^, R McWhinney^57^, J Porter^57^, M Purves^57^, L Snowball^57^, L Ferson^20^, M Gibson^20^, S Gillespie^20^, N Ignatius^20^, T Kane^20^, J Kwant^20,60,64^, M Matthews^20^, C McCallion^20^, C McConville^20^, M McDowell^20^, C McNally^20^, L Moore^20^, P Murphy^20^, A Nesbitt^20^, J Newell^20^, M Power^20^, E Reid^20^, K Robinson^20^, S Bacon^58^, N Booth^58^, A Bowring^58^, L Boxall^58^, J Burt^58^, J Cageao^58^, N Green^58^, K Gupwell^58^, S Keenan^58^, H Kingwell^58^, M Kryszkowska^58^, J Mortimore^58^, B Peace^58^, C Roughan^58^, T Allen^59^, G Dane^59^, K Harris^59^, S Hart^59^, SA Jones^59^, M Reader^59^, P Adair^60^, J Armstrong^60^, E Beggs^60^, I Bell^60^, C Edwards^60^, L Gilligan^60^, C Kelly^60^, M Kennedy^60^, J Kurian^60^, L Leal^60^, A McAtamney^60^, E McKay^60^, E Rogan^60^, M Smyth^60^, E Wiseman^60^, J Vahidassr^60^, E Bendix^61^, K Craig^61^, R Davison^61^, A Harrison^61^, A Smith^61^, K Ashton^62^, W Barkhuizen^62^, A Daniel^62^, C Dickinson^62^, H Durdu^62^, D Eastwood^62^, H Goddard^62^, R Hodkin^62^, J Howard^62^, C Jeffs^62^, S Joyce^62^, C Kelly^62^, G Kerr^62^, J Lanes^62^, B Magnall^62^, M McMahon^62^, M Moody^62^, S Patton^62^, R Taylor^62^, A Watson^62^, L Aird^63^, R Lakey^63^, J Murdy^63^, K Nelson^63^, G Storey^63^, R Brady^64^, D Holland^64^, M Kinnaird^64^, L Maltman^64^, D Martin^64^, K McCord^64^, S McKenna^64^, C Morgan^64^, C Shannon^64^, A Steele^64^, I Wiggam^64^, P Bagot^65^, D Baston^65^, C Bennett^65^, J Featherstone^65^, C Hare^65^, A McCluskey^65^, S Wade^65^, R Worton^65^, L Beale^66^, E Buckley^66^, K Byrne^66^, M Gasior^66^, B Robles^66^, C Smallwood^66^, S Stevens^66^, M Thomas^66^, V Williams^66^, S Armstrong^67^, V Brice^67^, A Edwards^67^, S Gething^67^, A Griffiths^67^, T Hills^67^, D Howells^67^, S Langdon^67^, S Moseley^67^, G Powell^67^, G Reynolds^67^, B Richard^67^, E Scott^67^, R White^67^, J Zebedee^67^, J Alexander^16^, L Brand^16^, E Colquhoun^16^, A Hill^16^, D Macartney^16^, H MacDonald^16^, B Manak^16^, H Morgan^16^, C Ritchie^16^, T Blair^68^, M Duffy^68^, J Graham^68^, J Scott^68^, T Vu^68^, P Yorston^68^, I Shakir^69^, C Button^69^, M Friend^69^, J Greig^69^, B Hairsine^69^, S Wade^69^, S Williamson^69^, T Adedoyin^70^, N Dayal^70^, S Gawned^70^, R Ghatala^70^, N Jeyaraj^70^, L Kerin^70^, L Montague^70^, C Orefo^70^, J O'Reilly^70^, J Styles^70^, S Trippier^70^, C Watchurst^70^, F Watson^70^, C Barrett^71^, J Cox^71^, F Hammonds^71^, K Quick^71^, K Robinson^71^, A Skinner^71^, C Vernon^71^, C Browne^72^, W Cameron^72^, V Coleman^72^, C Fulgencio^72^, L Gibson^72^, P Halliday^72^, D Heaney^72^, L Main^72^, K McGavin^72^, G Mead^72^, F Proudfoot^72^, A Redpath^72^, C Rodger^72^, S Scott^72^, L Baxter^74^, A Bryce^74^, M Halkett^74^, J Halliday^74^, A McAllister^74^, M McGuiness^74^, M Munro^74^, A Robb^74^, A Thompson^74^, B Tougher^74^, J Weadon^74^, J Young^74^, M McParland^75^, S Boyle^75^, B Byrne^75^, L Comiskey^75^, J Gilpin^75^, S Gilpin^75^, A Harris^75^, S Harshaw^75^, J Haughey^75^, F McArdle^75^, L McConnell^75^, E McEneaney^75^, M Millar^75^, M Murphy^75^, J Tilley^75^.

^1^Deakin Health Economics, Institute for Health Transformation, Deakin University, Geelong, Australia. ^2^Florey Institute of Neuroscience and Mental Health, University of Melbourne, Heidelberg, Australia. ^3^Eastern Health Clinical School, Monash University, Box Hill, Australia. ^4^Austin Hospital, Melbourne, Australia. ^5^University of Glasgow, Glasgow, United Kingdom. ^6^University of Sydney, Sydney, Australia. ^7^Monash University, Melbourne, Australia. ^8^Norwegian University of Science and Technology, Trondheim, Norway. ^9^University of Nottingham, Nottingham, United Kingdom. ^10^Box Hill Hospital, Melbourne, Australia. ^11^Royal Brisbane and Women’s Hospital, Melbourne, Australia. ^12^Austin Health, Melbourne, Australia. ^13^Australian Catholic University, Sydney, Australia. ^14^Alfred Hospital, Melbourne, Australia. ^15^Monash Health, Melbourne, Australia. ^16^Western Infirmary, Glasgow, United Kingdom. ^17^University of Southampton, Southampton, United Kingdom. ^18^Newcastle University, Newcastle upon Tyne, United Kingdom. ^19^University of Ulster, Belfast, United Kingdom. ^20^Ulster Hospital, Belfast, United Kingdom. ^21^Singapore General Hospital, Singapore, Singapore. ^22^Gothenberg University, Gothenburg, Sweden. ^23^University of Central Lancashire, Preston, United Kingdom. ^24^Unaffilated. ^25^Royal Melbourne Hospital, Melbourne, Australia. ^26^Royal Perth Hospital, Perth, Australia. ^27^Frankston Hospital, Melbourne, Australia. ^28^Westmead Hospital, Sydney, Australia. ^29^Geelong Hospital, Geelong, Australia. ^30^Flinders Medical Centre, Adelaide, Australia. ^31^Western Hospital, Melbourne, Australia. ^32^Epworth Hospital, Melbourne, Australia. ^33^St George Hospital, Sydney, Australia. ^34^Albury Hospital, Albury, Australia. ^35^Nambour General Hospital, Nambour, Australia. ^36^Sir Charles Gairdner Hospital, Perth, Australia. ^37^Warrnambool Base Hospital, Warrnambool, Australia. ^38^West Gippsland Hospital, Warragul, Australia. ^39^St Vincent’s Hospital, Sydney, Australia. ^40^Wyong Public Hospital, Wyong, Australia. ^41^The Wesley Hospital, Brisbane, Australia. ^42^Calvary Mater Newcastle Hospital, Newcastle, Australia. ^43^Wodonga Hospital, Wodonga, Australia. ^44^Belmont Hospital, Perth, Australia. ^45^Wollongong Hospital, Wollongong, Australia. ^46^Gosford Hospital, Gosford, Australia. ^47^Auckland City Hospital, Auckland, New Zealand. ^48^UKM Medical Centre, Kuala Lumpur, Malaysia. ^49^Forth Valley Royal Hospital, Larbert, United Kingdom. ^50^Yeovil District Hospital, Yeovil, United Kingdom. ^51^York Hospital, York, United Kingdom. ^52^Royal Victoria Infirmary, Newcastle upon Tyne, United Kingdom. ^53^Aberdeen Royal Infirmary, Aberdeen, United Kingdom. ^54^Royal Bournemouth Hospital, Bournemouth, United Kingdom. ^55^Imperial College Healthcare, St Mary’s Hospital, London, United Kingdom. ^56^Wishaw General Hospital, Wishaw, United Kingdom. ^57^Monklands Hospital, Airdie, United Kingdom. ^58^Royal Devon and Exeter Hospital, Exeter, United Kingdom. ^59^Queen Elizabeth The Queen Mother Hospital, Margate, United Kingdom. ^60^Antrim Area Hospital, Antrim, United Kingdom. ^61^Wansbeck General Hospital, Ashington, United Kingdom. ^62^Blackpool Hospital, Blackpool, United Kingdom. ^63^North Tyneside General Hospital, North Shields, United Kingdom. ^64^Belfast City Hospital, Belfast, United Kingdom. ^65^Harrogate District Hospital, Harrogate, United Kingdom. ^66^St Mary’s Hospital, Isle of Wight, Newport, United Kingdom. ^67^Nevill Hall Hospital, Abergavenny, United Kingdom. ^68^South Tyneside District Hospital, South Shields, United Kingdom. ^69^Calderdale Royal Hospital, Halifax, United Kingdom. ^70^St George’s Hospital, London, United Kingdom. ^71^North Devon District Hospital, Barnstaple, United Kingdom. ^72^Royal Infirmary of Edinburgh, Edinburgh, United Kingdom. ^73^Hexham General Hospital, Hexham, United Kingdom. ^74^University Hospital Crosshouse, Crosshouse, United Kingdom. ^75^Daisy Hill Hospital, Newry, United Kingdom.
